# Supplementary material for: The π-trap approach for obtaining crystal structure data of inherently amorphous cluster compounds
Source: Nat Commun. 2025 Aug 25;16:7903. doi: 10.1038/s41467-025-62928-y (PMC12378380; doi:10.1038/s41467-025-62928-y)
Supplement: Supplementary file 1 — Supplementary Information [file 41467_2025_62928_MOESM1_ESM.pdf]

# Supporting Information

## The π-Trap Approach for Obtaining Crystal Structure Data of Inherently Amorphous Cluster Compounds

Yaofeng Wang[1], Niklas Rinn[1], Kevin Eberheim[2], Ferdinand Ziese[2], Jan Christmann[1], Arijit Jana,[1] Simon Nier,[1] Nils W. Rosemann,[1] Simone Sanna[2], and Stefanie Dehnen[1,*]

[1] Karlsruhe Institute of Technology, Institute of Nanotechnology. Kaiserstrasse 12, 76131 Karlsruhe, Germany

[2] Institut für Theoretische Physik and Center for Materials Research (LaMa), Justus-Liebig-Universität Gießen, 35392 Gießen, Germany

*Corresponding author(s). E-mail(s): stefanie.dehnen@kit.edu;

Contents

1. **Supplementary information of the X-ray diffraction analyses of single-crystalline compounds 1 – 6 ..... 2**
2. **Supplementary information on the optical absorption spectroscopy ..... 16**
3. **Supplementary information on the nonlinear optical response ..... 17**
4. **Supplementary information on the first-principles calculations of the electronic properties of the cocrystals..... 18**
5. **References..... 24**

## 1. Supplementary information of the X-ray diffraction analyses of single-crystalline compounds 1 – 6

**Supplementary Table 1 | Crystallographic data and details of the structure determinations of compound 1 – 6.**

| Compound | $[(PhSn)_4S_6]_2{\cdot}(C_{60}){\cdot}(C_7H_8)_{1.2}{\cdot}(C_4H_8O)_{1.2}$ (**1**) | $[(PhSn)_4S_6]_2{\cdot}(C_{60})_{1.5}{\cdot}(C_7H_8)$ (**2**) | $[(PhSn)_4Se_6]{\cdot}(C_{60}){\cdot}(C_7H_8){\cdot}(C_4H_8O)_{0.5}$ (**3**) |
|---|---|---|---|
| Chemical formula | $C_{121.2}H_{59.2}O_{1.2}S_{12}Sn_8$ | $C_{145}H_{48}S_{12}Sn_8$ | $C_{93}H_{32}O_{0.5}Se_6Sn_4$ |
| Formula weight /[g mol$^{-1}$] | 2868.72 | 3124.07 | 2105.70 |
| Temperature/K | 150 | 150 | 180 |
| Crystal system | trigonal | monoclinic | orthorhombic |
| Space group | $R\overline{3}$ | $P2_1/c$ | *Pbnb* |
| a/Å | 27.2073(14) | 23.032(4) | 18.6432(11) |
| b/Å | 27.2073(14) | 19.248(3) | 25.1311(15) |
| c/Å | 35.229(2) | 24.378(5) | 28.9919(17) |
| α/° | 90 | 90 | 90 |
| β/° | 90 | 102.013(15) | 90 |
| γ/° | 120 | 90 | 90 |
| Volume/Å$^3$ | 22584(3) | 10565.5(15) | 13583.4(14) |
| Z | 9 | 4 | 8 |
| $\rho_{calc}$g/cm3 | 1.898 | 1.964 | 2.059 |
| μ/mm-1 | 12.306 | 11.895 | 10.626 |
| F(000) | 12492.0 | 6040.0 | 7984.0 |
| Crystal size/mm$^3$ | 0.2 × 0.15 × 0.1 | 0.06 × 0.04 × 0.02 | 0.1 × 0.1 × 0.05 |
| Radiation | Ga Kα (λ = 1.34143) | Ga Kα (λ = 1.34143) | Ga Kα (λ = 1.34143) |
| 2Θ range for data collection/° | 3.926 to 104.996 | 5.136 to 111.766 | 5.78 to 111.432 |
| Index ranges | $-32 \le h \le 32$, $-23 \le k \le 32$, $-41 \le l \le 39$ | $27 \le h \le 28$, $-23 \le k \le 21$, $-29 \le l \le 19$ | $-18 \le h \le 22$, $-28 \le k \le 30$, $-35 \le l \le 23$ |
| Reflections collected | 48201 | 63933 | 50996 |
| Independent reflections | 8688 [$R_{int}$ = 0.1291, $R_{sigma}$ = 0.1095] | 19768 [$R_{int}$ = 0.0430, $R_{sigma}$ = 0.0603] | 12910 [$R_{int}$ = 0.0709, $R_{sigma}$ = 0.1413] |
| Goodness-of-fit on F$^2$ | 1.030 | 1.026 | 0.716 |
| Final R indexes [I ⩾ 2σ (I)] | $R_1$ = 0.0734, $wR_2$ = 0.01846 | $R_1$ = 0.0562, $wR_2$ = 0.1305 | $R_1$ = 0.0308, $wR_2$ = 0.0499 |
| Final R indexes [all data] | $R_1$ = 0.1175, $wR_2$ = 0.1972 | $R_1$ = 0.1034, $wR_2$ = 0.1604 | $R_1$ = 0.0774, $wR_2$ = 0.0533 |
| Largest diff. peak/hole / e Å$^{-3}$ | 1.56/-1.15 | 1.71/-1.92 | 0.97/-0.54 |
| CCDC deposition number | 2419991 | 2419992 | 2419993 |

## Supplementary Table 1 (continued)

| Compound | $[(NpSn)_4S_6]_2\cdot(C_{60})$ (**4**) | $[(NpSn)_4S_6]\cdot(C_{70})\cdot(C_7H_8)$ (**5**) | $[(NpSn)_4S_6]_4\cdot[Lu_3N@C_{80}]$ (**6**) |
|---|---|---|---|
| Chemical formula | $C_{140}H_{56}S_{12}Sn_8$ | $C_{120.5}H_{40}S_6Sn_4$ | $C_{240}H_{112}Lu_3NS_{24}Sn_{16}$ |
| Formula weight /[g $mol^{-1}$] | 3072.08 | 2154.64 | 6202.69 |
| Temperature/K | 150 | 100 | 150 |
| Crystal system | triclinic | triclinic | triclinic |
| Space group | $P\overline{1}$ | $P\overline{1}$ | $P\overline{1}$ |
| a/Å | 12.3156(11) | 13.4243(5) | 12.9100(16) |
| b/Å | 12.8584(12) | 16.6129(6) | 12.8581(16) |
| c/Å | 17.4279(17) | 20.1458(8) | 18.360(2) |
| α/° | 94.357(8) | 67.359(3) | 83.237(10) |
| β/° | 101.346(7) | 81.621(3) | 70.276(10) |
| γ/° | 101.093(7) | 72.552(3) | 70.550(9) |
| Volume/$Å^3$ | 2636.7(4) | 3953.5(3) | 2705.2(6) |
| Z | 1 | 2 | 0.5 |
| $\rho_{calc}g/cm^3$ | 1.935 | 1.810 | 1.904 |
| $\mu/mm^{-1}$ | 11.905 | 8.086 | 13.213 |
| F(000) | 1488.0 | 2118.0 | 1478.0 |
| Crystal size/$mm^3$ | 0.1 × 0.05 × 0.1 | 0.1 × 0.05 × 0.03 | 0.15 × 0.01 × 0.01 |
| Radiation | Ga Kα (λ = 1.34143) | Ga Kα (λ = 1.34143) | Ga Kα (λ = 1.34143) |
| 2Θ range for data collection/° | 6.138 to 118.316 | 5.35 to 104 | 6.342 to 112.226 |
| Index ranges | $-15 \leq h \leq 8$, $-15 \leq k \leq 16$, $-20 \leq l \leq 22$ | $-15 \leq h \leq 15$, $-18 \leq k \leq 19$, $-23 \leq l \leq 19$ | $-15 \leq h \leq 15$, $-15 \leq k \leq 13$, $-22 \leq l \leq 18$ |
| Reflections collected | 26939 | 85280 | 38490 |
| Independent reflections | 10921 [$R_{int}$ = 0.0253, $R_{sigma}$ = 0.0338] | 13349 [Rint = 0.1724, Rsigma = 0.1106] | 10385 [$R_{int}$ = 0.0677, $R_{sigma}$ = 0.1850] |
| Goodness-of-fit on $F^2$ | 1.010 | 0.867 | 0.765 |
| Final R indexes [I>=2σ (I)] | $R_1$ = 0.0335, $wR_2$ = 0.0857 | $R_1$ = 0.0494, $wR_2$ = 0.1161 | $R_1$ = 0.0389, $wR_2$ = 0.0868 |
| Final R indexes [all data] | $R_1$ = 0.0426, $wR_2$ = 0.0881 | $R_1$ = 0.1056, $wR_2$ = 0.1297 | $R_1$ = 0.0996, $wR_2$ = 0.0930 |
| Largest diff. peak/hole / e $Å^{-3}$ | 1.90/-1.62 | 1.57/-1.08 | 1.28/-0.91 |
| CCDC deposition number | 2456507 | 2456508 | 2456509 |

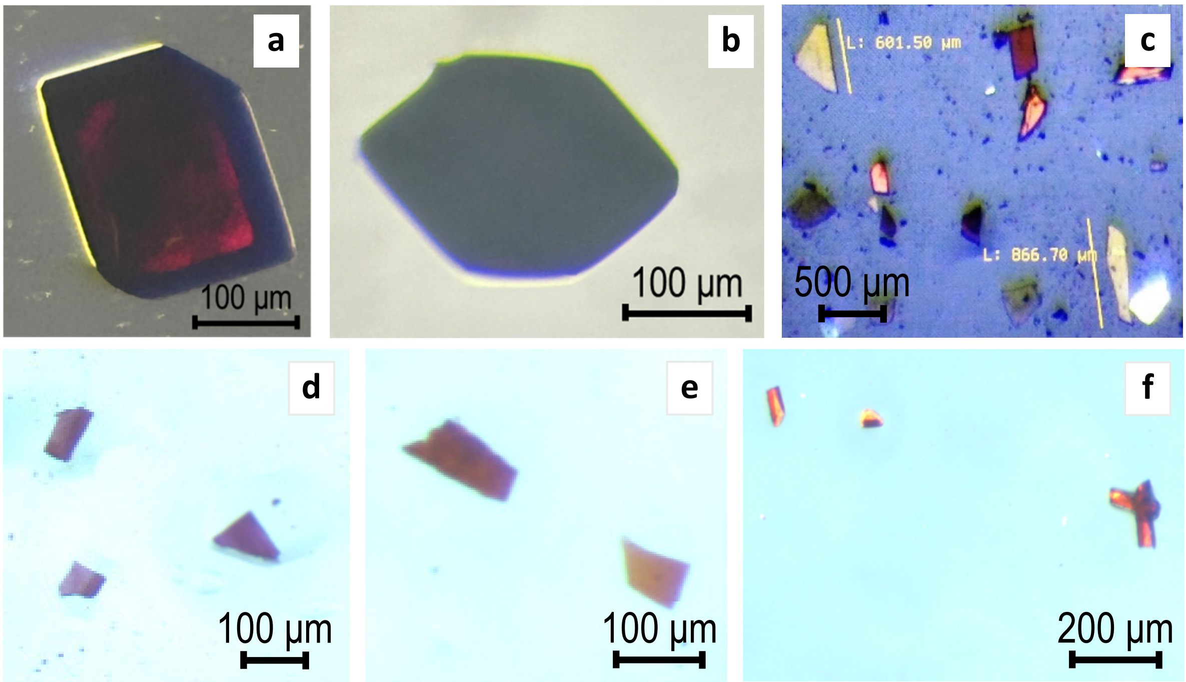


**Supplementary Figure 1 | Optical photograph of crystals of compounds 1 - 6. a,** Compound **1**. **b,** Compound **2**. **c,** Compound **3**. **d,** Compound **4**. **e,** Compound **5**. **f,** Compound **6**.

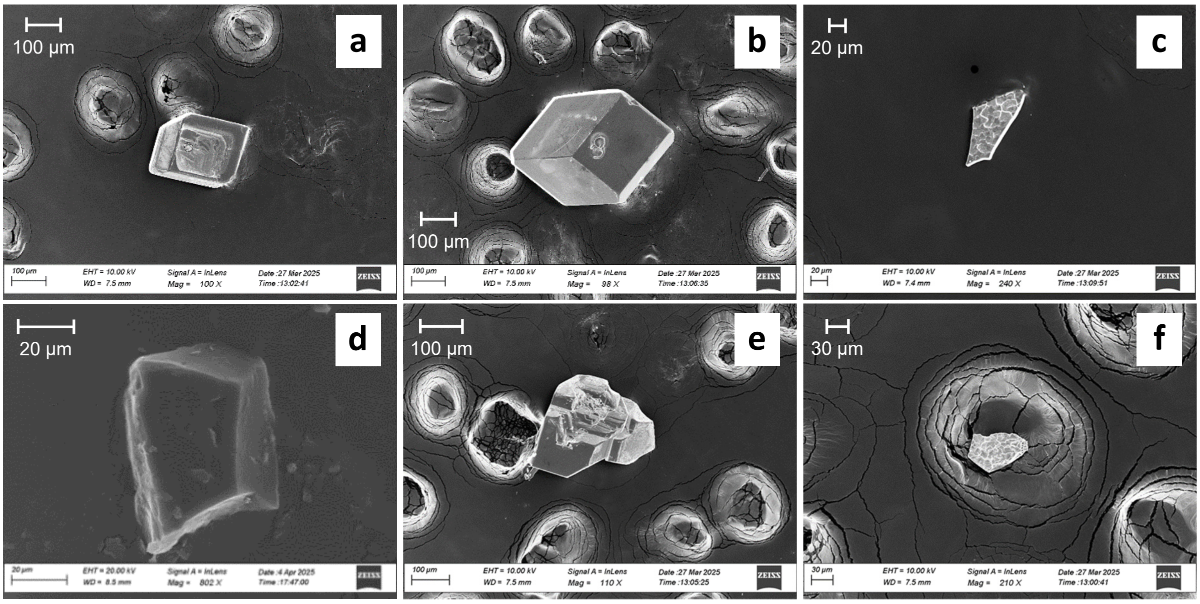


**Supplementary Figure 2 | SEM images of crystals of compounds 1 - 6. a,** Compound **1**. **b,** Compound **2**. **c,** Compound **3**. **d,** Compound **4**. **e,** Compound **5**. **f,** Compound **6**.

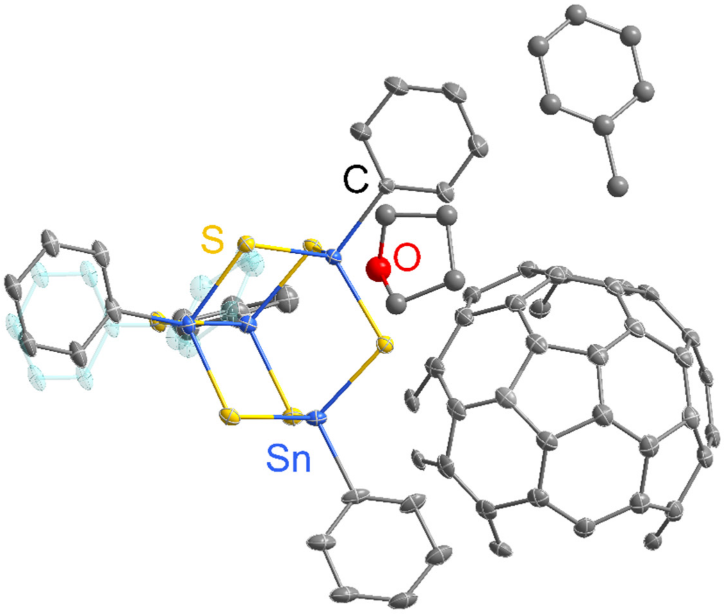


**Supplementary Figure 3 | Asymmetric unit of the crystal structure of compound 1 (CCDC-2419991).** Ellipsoids are shown at 30% probability. The disorder of the phenyl groups is shown as transparent atoms. H atoms are omitted for clarity.

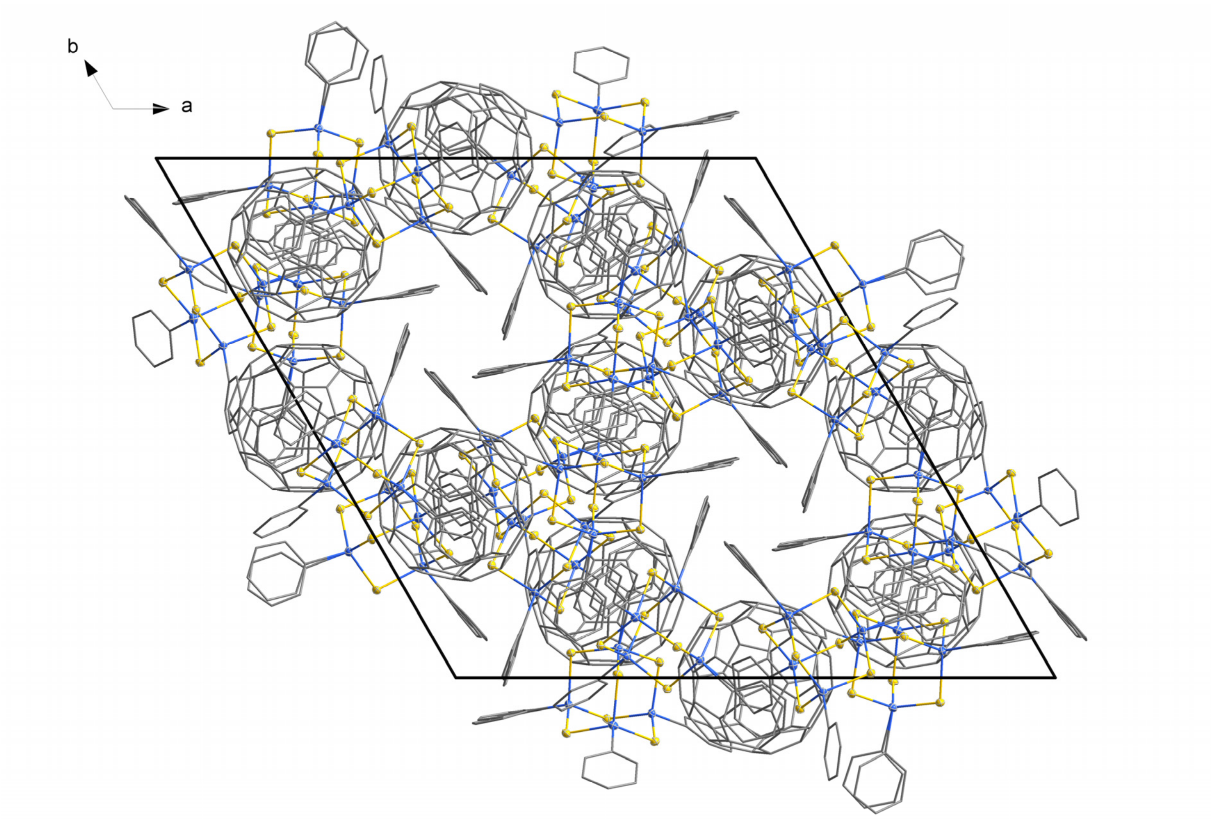


**Supplementary Figure 4 | Extended unit cell of the crystal structure of 1 viewed along the crystallographic c-axis.** Ellipsoids are shown at 30% probability. Solvent molecules and H atoms are omitted for clarity.

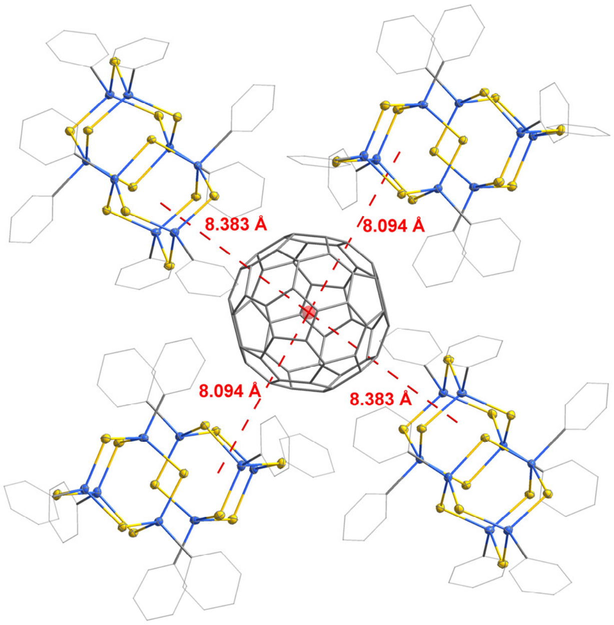


**Supplementary Figure 5 | Cutout of the crystal structure of 1, showing the surrounding of each $C_{60}$ molecule and their interaction with surrounding [$(PhSn)_4S_6$] pairs.** Ellipsoids are shown at 30% probability. The disorder of the phenyl groups is omitted for clarity, only the major component of the disordered parts is shown.

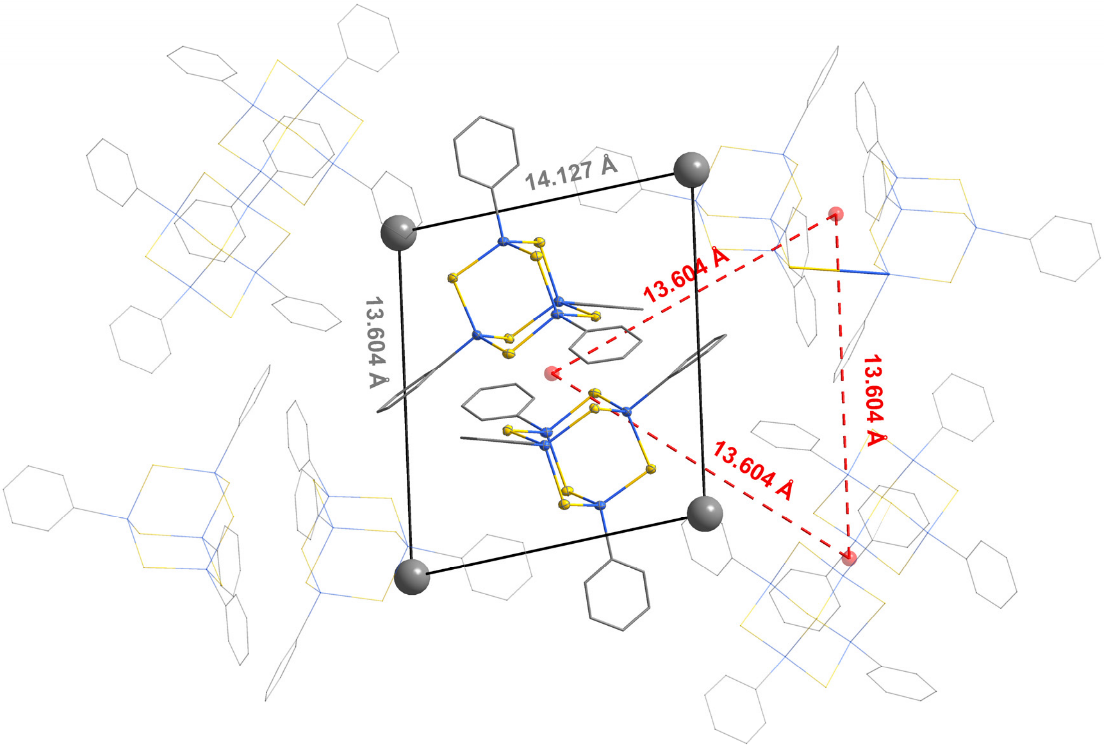


**Supplementary Figure 6 | The [$(PhSn)_4S_6$] pair in the crystal structure of compound 1.** The figure illustrates the interaction of [$(PhSn)_4S_6$] molecules with surrounding $C_{60}$ molecules (here simplified as their centroids as grey spheres) and adjacent [$(PhSn)_4S]_6$ pairs. Ellipsoids are shown at 30% probability.

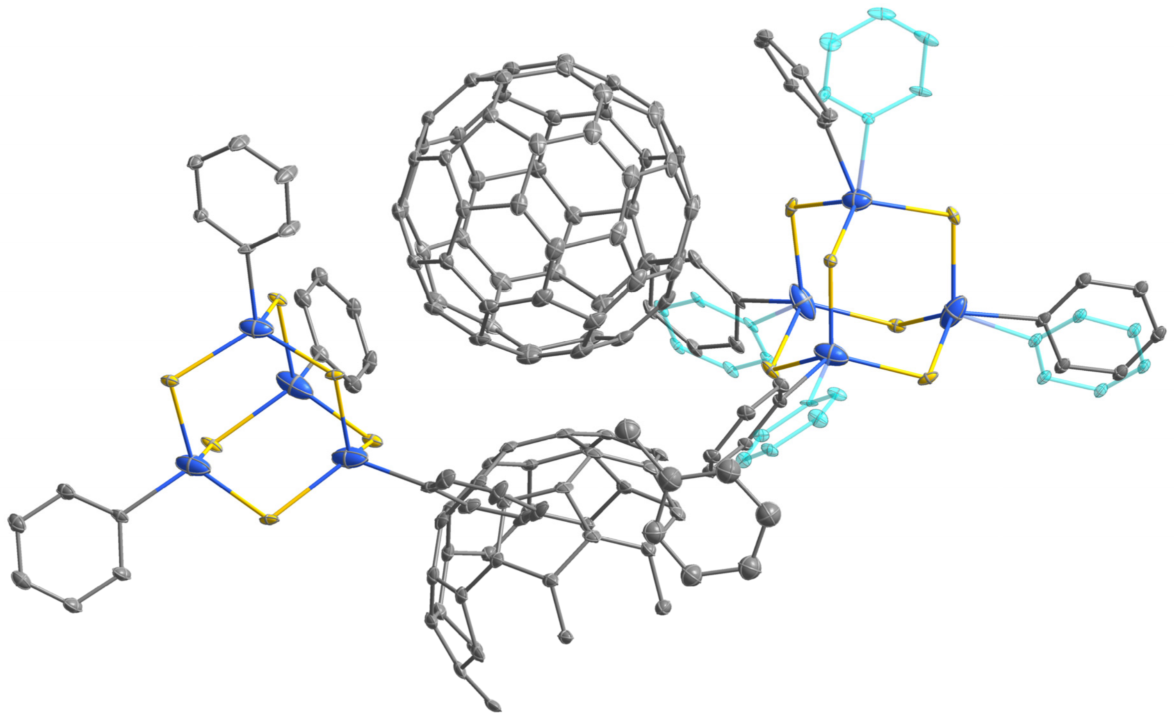

**Supplementary Figure 7 | Asymmetric unit of the crystal structure of 2 (CCDC-2419992).** Ellipsoids are shown at 30% probability. The disorder in the positions of the phenyl groups is shown as transparent atoms. H atoms are omitted for clarity.

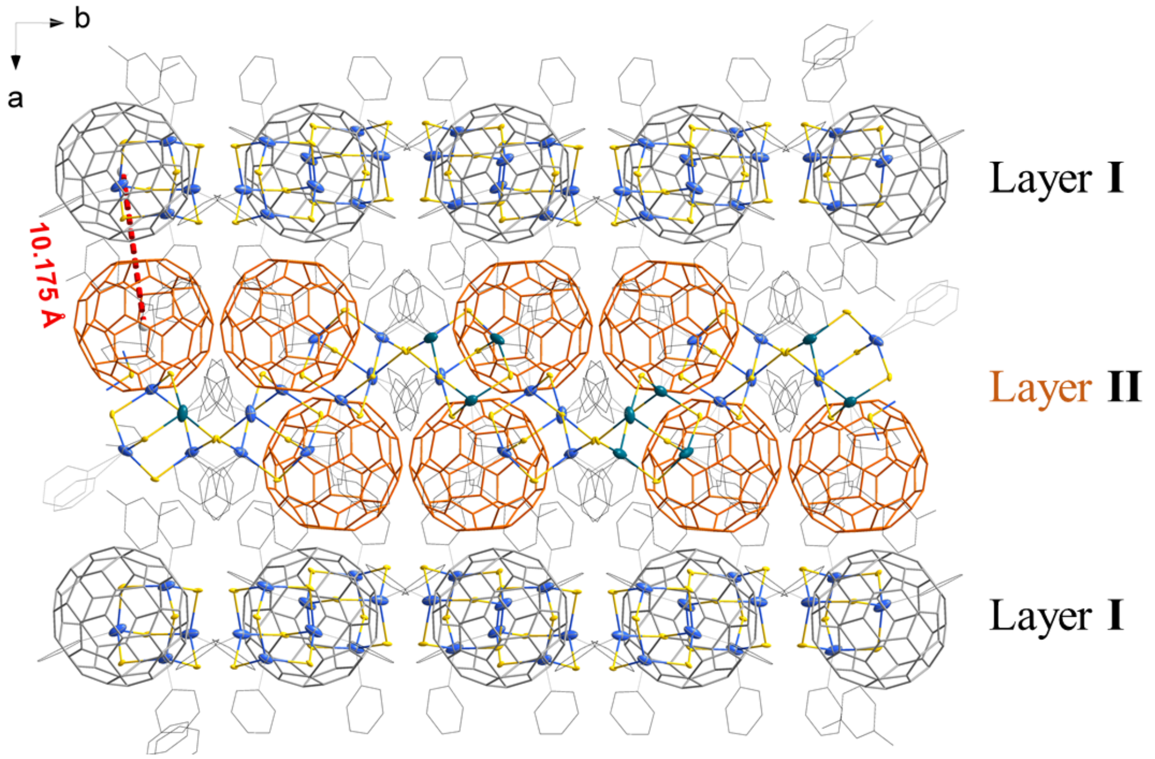


**Supplementary Figure 8 | Packing of the molecules in 2 viewed along the crystallographic c-axis.** Ellipsoids are shown at 30% probability. Hydrogen atoms are omitted for clarity.

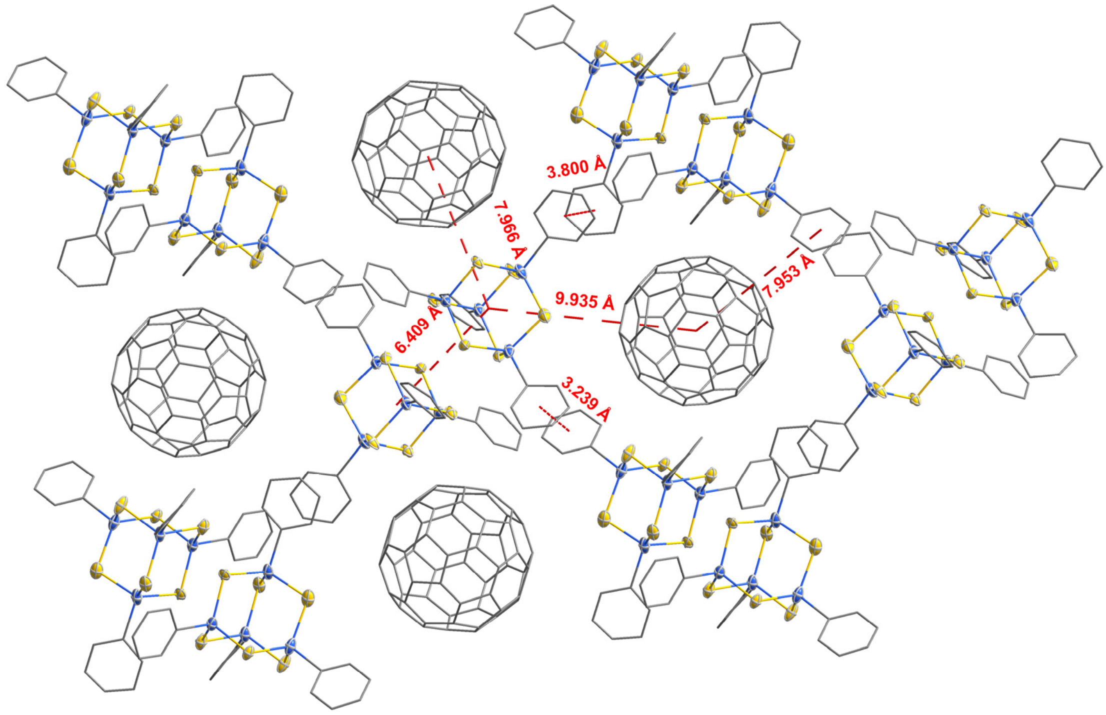


**Supplementary Figure 9 | Packing of the molecules in layer I of the crystal structure of compound 2 viewed along the crystallographic a-axis.** Ellipsoids are shown at 30% probability.

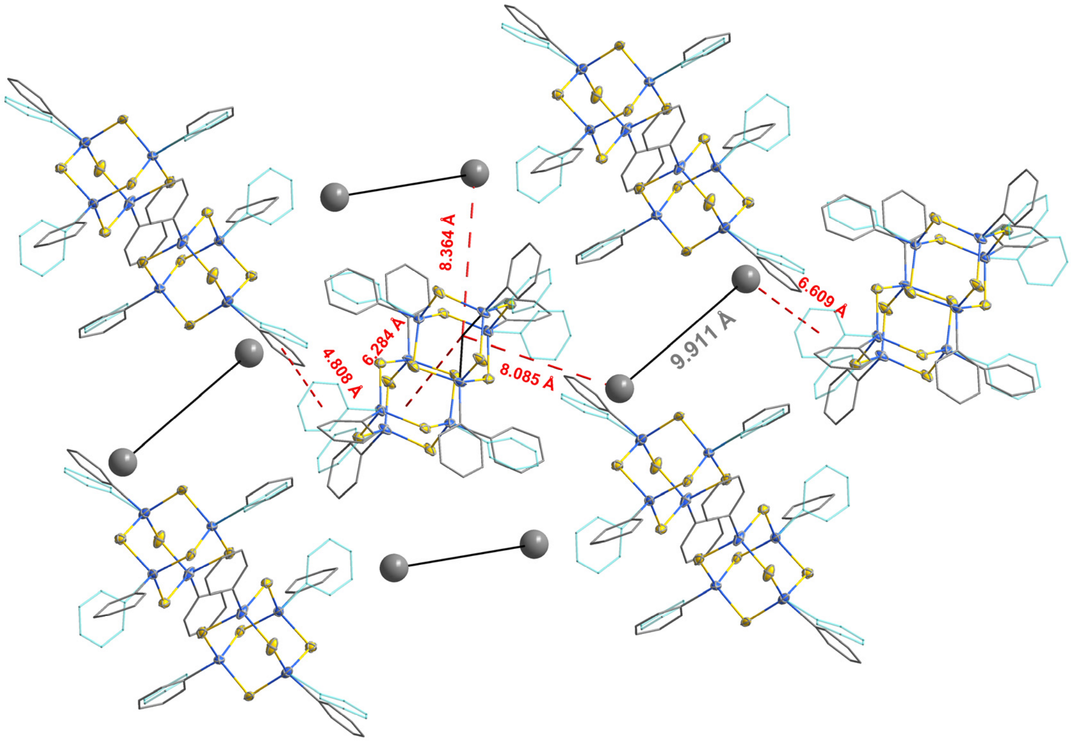


**Supplementary Figure 10 | Packing of the molecules in layer II of the crystal structure of compound 2 viewed along the crystallographic a-axis.** $C_{60}$s are only shown as grey spheres at their centroid positions. Ellipsoids are shown at 30% probability.

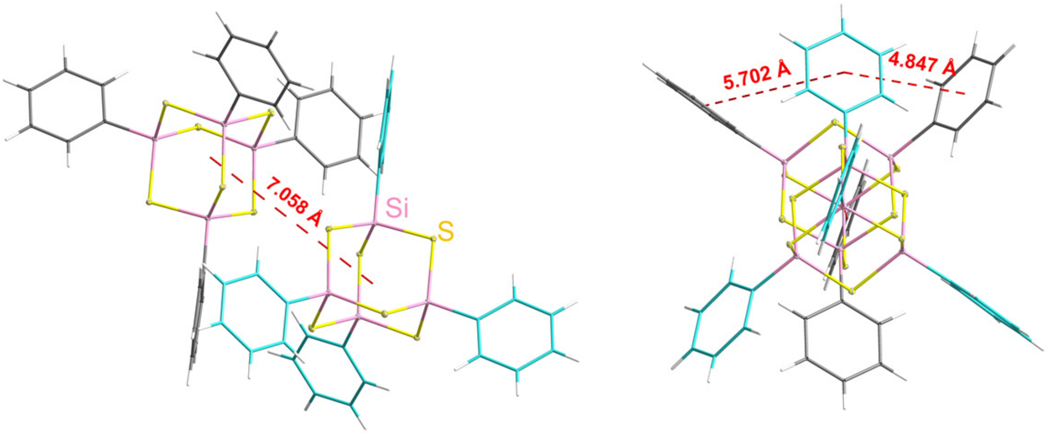


**Supplementary Figure 11 | Cluster pair in the crystal structure of [(PhSi)$_4$S$_6$] (C)[1] (two views) for comparison.** Ellipsoids are shown at 30% probability. The phenyl groups are depicted in grey and teal for the two distinct [(PhSi)$_4$S$_6$] molecules within the pair, respectively.

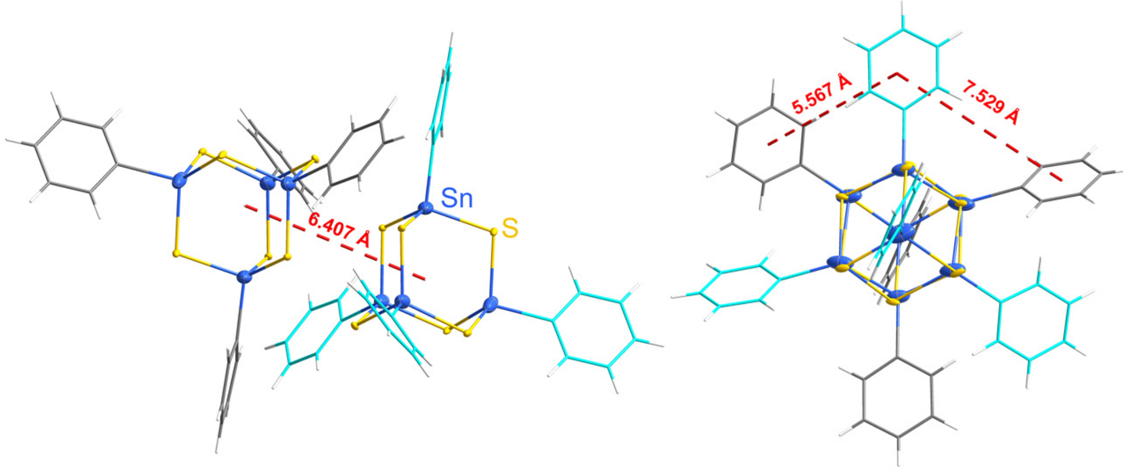


**Supplementary Figure 12 | Orientation of the [(PhSn)$_4$S$_6$] pair in layer I of the crystal structure of 2 (two views).** Ellipsoids are shown at 30% probability. The phenyl groups are depicted in grey and teal for the two distinct [(PhSn)$_4$S$_6$] molecules within the pair, respectively.

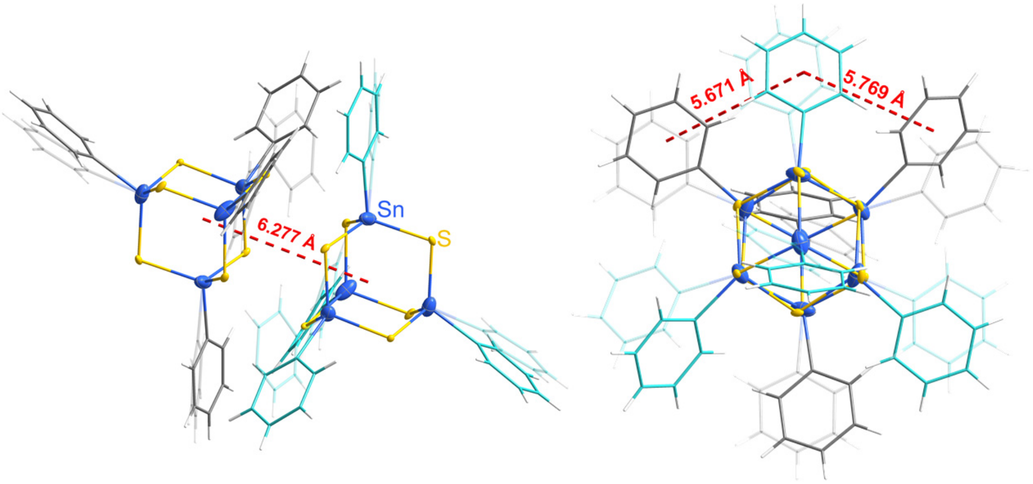


**Supplementary Figure 13 | The orientation of the [(PhSn)$_4$S$_6$] pairs in layer II of the crystal structure of 2 (two views).** The phenyl groups are depicted in grey and teal for the two distinct [(PhSn)$_4$S$_6$] molecules within the pair, respectively. Ellipsoids are shown at 30% probability.

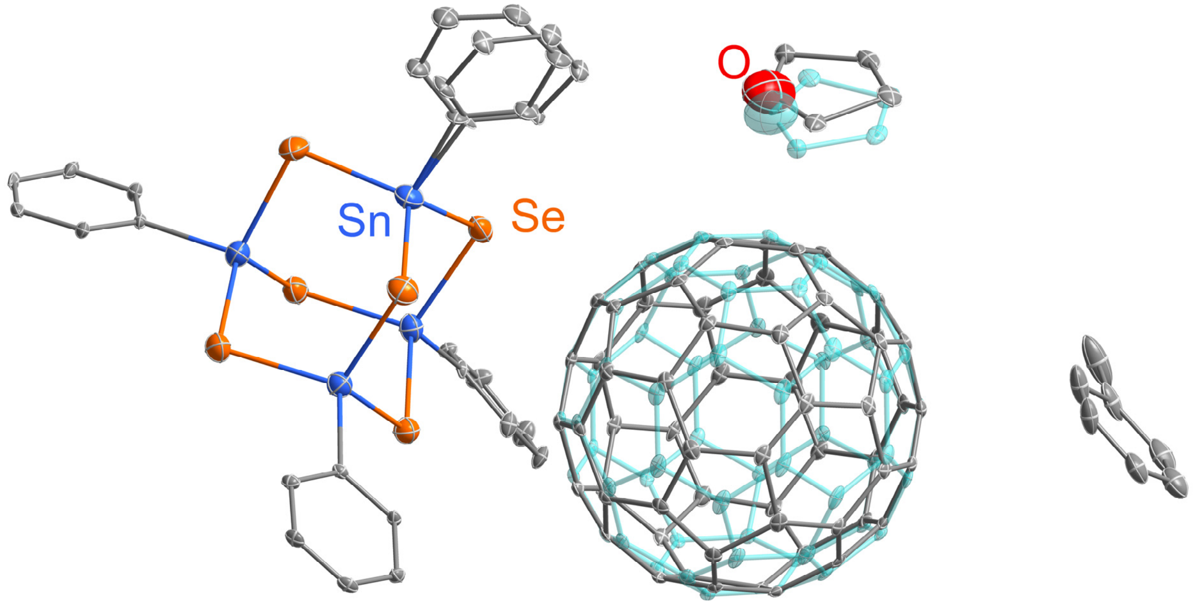


**Supplementary Figure 14 | Asymmetric unit of the crystal structure of 3 (CCDC-2419993).** Ellipsoids are shown at 30% probability. The disorder of the phenyl groups is shown as transparent atoms. H atoms are omitted for clarity.

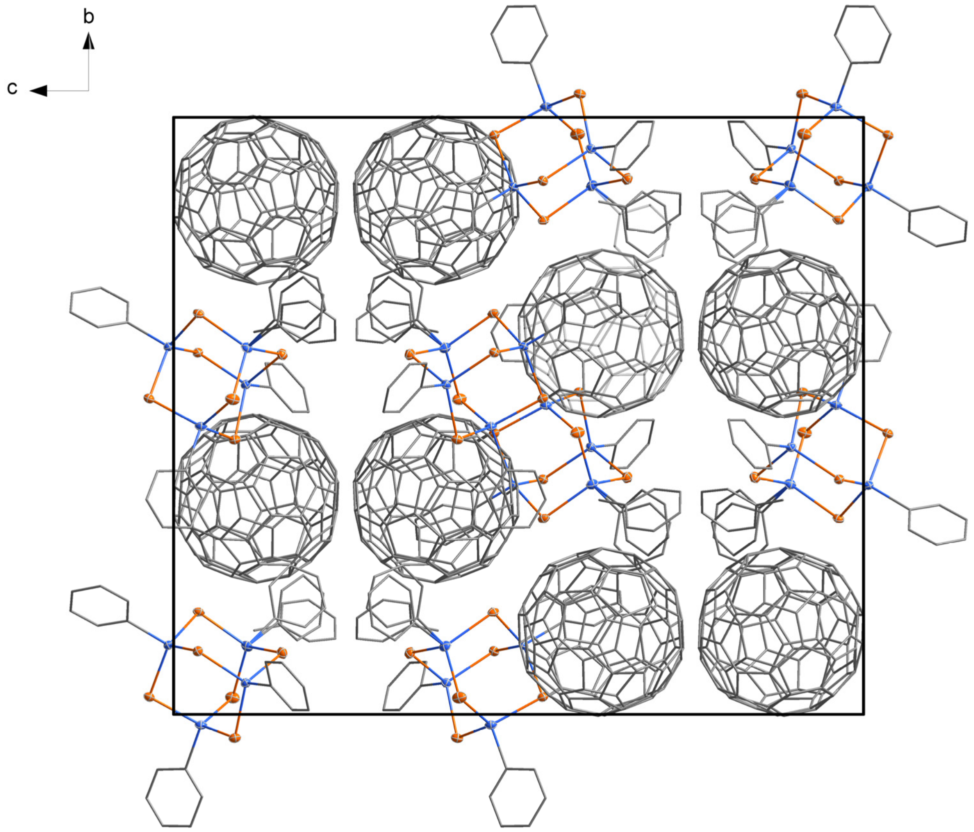


**Supplementary Figure 15 | Extended unit cell of the crystal structure of 3 viewed along the crystallographic a-axis.** Ellipsoids are shown at 30% probability. Solvents are masked with Olex2 mask function and H atoms are omitted for clarity.

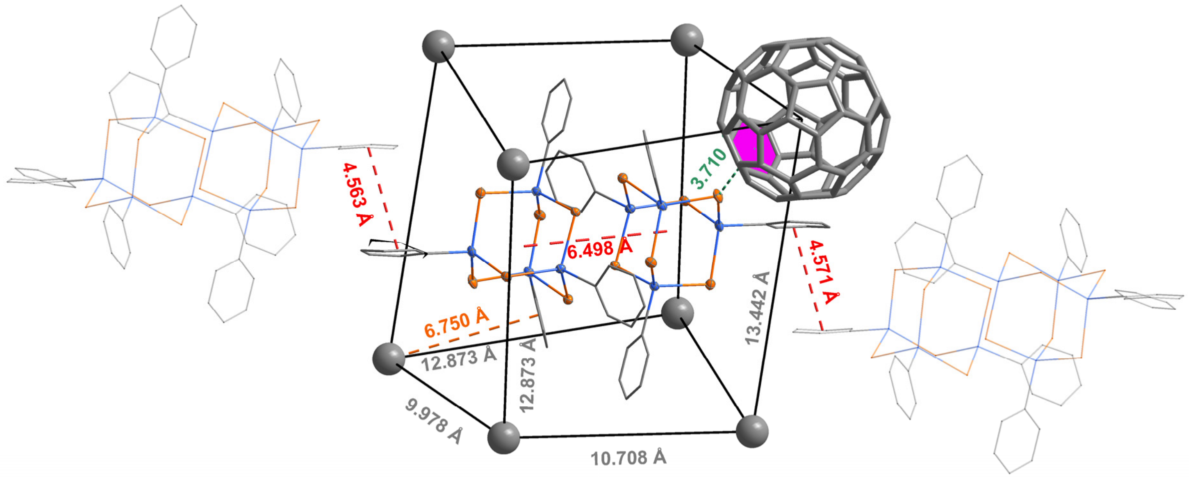


**Supplementary Figure 16 | Cutout from the structure of 3.** The figure illustrates the interactions of $[(PhSn)_4Se_6]$ molecules with surrounding $C_{60}$ molecules (here simplified as their centroids as grey spheres) and adjacent $[(PhSn)_4Se_6]$ pairs. Ellipsoids are shown at 30% probability.

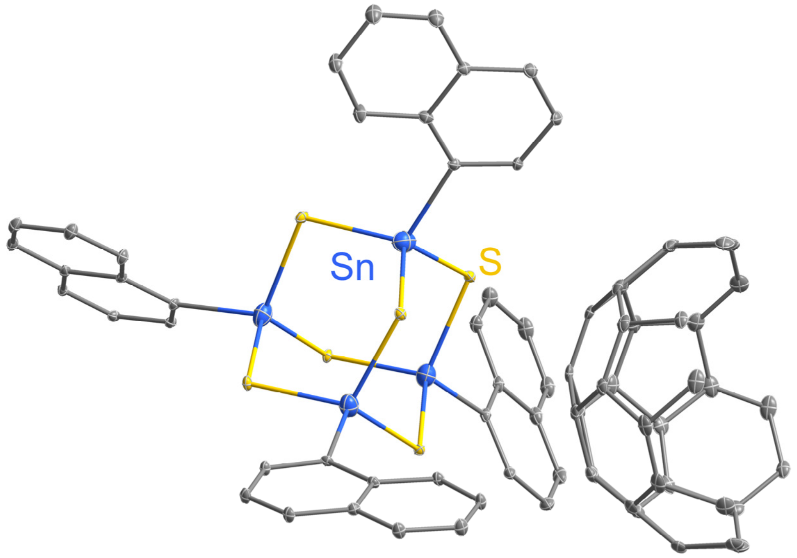


**Supplementary Figure 17 | Asymmetric unit of the crystal structure of 4 (CCDC-2456507).** Ellipsoids are shown at 30% probability. H atoms are omitted for clarity.

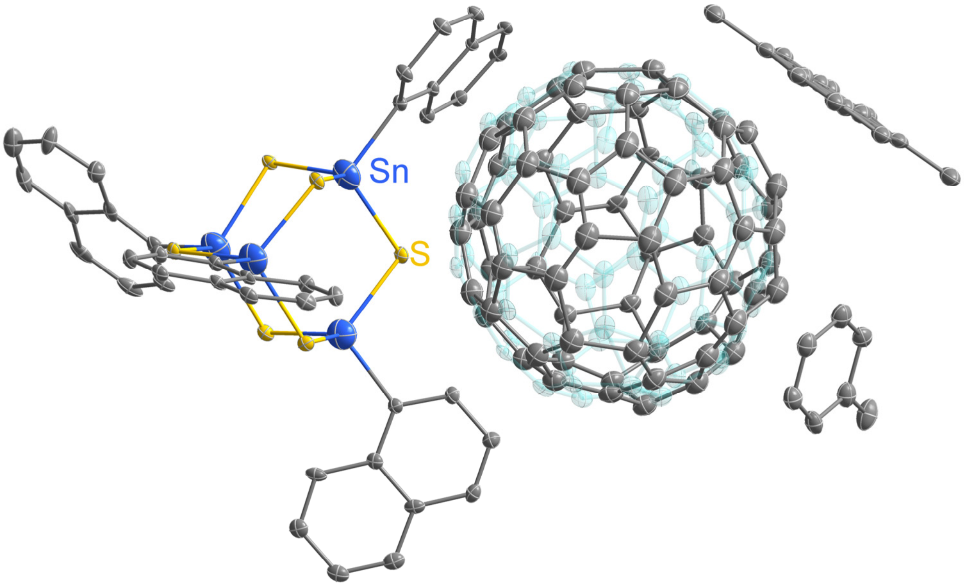


**Supplementary Figure 18 | Asymmetric unit of the crystal structure of 5 (CCDC-2456508).** Ellipsoids are shown at 30% probability. The disorder of the phenyl groups is shown as transparent atoms. H atoms are omitted for clarity.

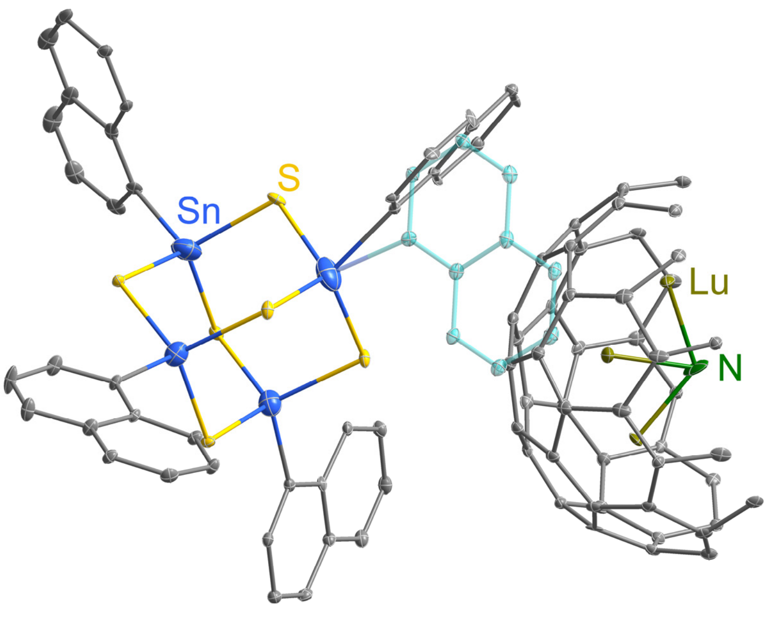


**Supplementary Figure 19 | Asymmetric unit of the crystal structure of 6 (CCDC-2456509).** Ellipsoids are shown at 30% probability. The disorder of the phenyl groups is shown as transparent atoms. H atoms are omitted for clarity.

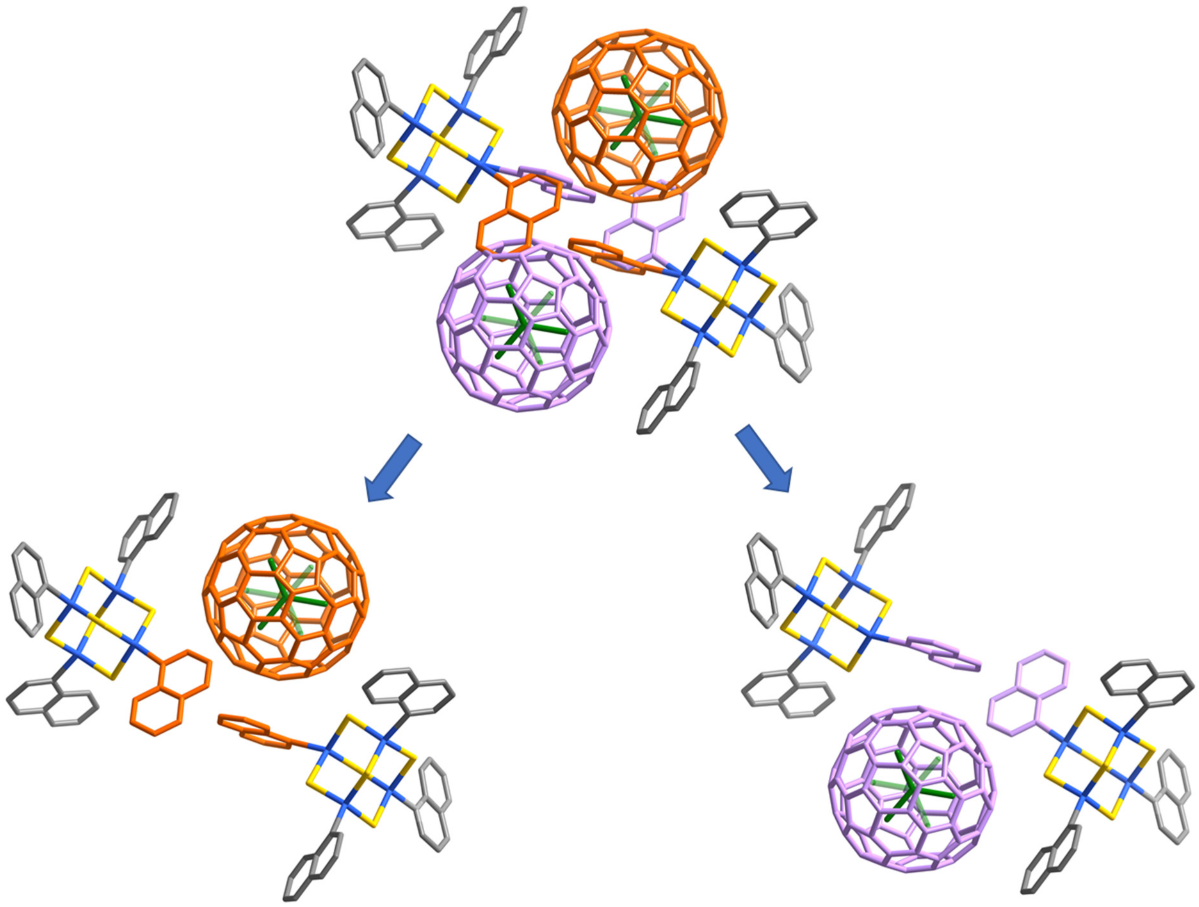

**Supplementary Figure 20 | Asymmetric units of the crystal structure of 6 with statistical disorder of one fullerene molecule and two nearest naphthyl substituents.** The two possible orientations of the involved naphthyl substituents and the fullerene molecule with a site occupation factor (s.o.f.) of 0.5 are highlighted in orange or violet color, respectively. The different orientations of the naphthyl groups that accompany the different position of the fullerene molecule underline the importance of the secondary interactions between the two components in the cocrystal. The rotational disorder of the $Lu_3N$ groups is shown as transparent atoms. H atoms are omitted for clarity.

**Supplementary Table 2 | Selected bonds length and angles of [$(PhSn)_4S_6$] in compounds 1 and 2, [$(PhSn)_4Se_6$] in compound 3, and [$(PhSi)_4S_6$] in compound C (ref [1]) for comparison.**

| Compound | Bond distance / Angle | |
|---|---|---|
| [$(PhSn)_4S_6$] in **1** | Sn–S<br>Sn–C<br>Sn…Sn<br>S…S | 2.395(4) – 2.411(3) Å<br>2.103(14) –2.120(17) Å<br>3.7631(13) –3.8105(18) Å<br>3.9139(48) – 3.9727(60) Å |
| | S–Sn–S<br>Sn–S–Sn | 108.95(13) – 116.00(12) °<br>103.49(13) –105.31(13) ° |
| [$(PhSn)_4S_6$] in **2** | Sn–S<br>Sn–C<br>Sn…Sn<br>S…S | 2.38(1) –2.411(9) Å<br>2.105(12) –2.166(17) Å<br>3.7427(12) –3.8287(12) Å<br>3.8753(44) – 4.0789(44) Å |
| | S–Sn–S<br>Sn–S–Sn | 107.8(4) – 116.0(3) °<br>103.09(13) – 104.76(15) ° |
| [$(PhSn)_4Se_6$] in **3** | Sn–Se<br>Sn–C<br>Sn…Sn<br>S…S | 2.5135(7) –2.5337(7) Å<br>2.11(2) –2.126(19) Å<br>3.8952(20) – 3.9242(10) Å<br>4.1581(37) –4.2870(39) Å |
| | Se–Sn–Se<br>Sn–S–Sn | 110.50(2) – 116.24(2) °<br>100.81(8) – 102.36(8) ° |
| [$(PhSi)_4S_6$] in **C** [ref. 1] | Si–S<br>Si–C<br>Si…Si<br>S…S | 2.1184(5) – 2.1476(5) Å<br>1.8540(15) –1.8562(15) Å<br>3..3393(5) – 3.3676(6) Å<br>3.5237(5) – 3.5638(5) Å |
| | S–Si–S<br>S–S–Si | 111.29(2) – 113.27(2) °<br>103.22(2) – 104.25(2) ° |

**Supplementary Table 3 | Selected bonds length and angles of [$(NpSn)_4S_6$] in compounds 4 -6, and [$(NpSi)_4S_6$] in compound D (ref. [2]) for comparison.**

| Compound | Bond distance / Angle | |
|---|---|---|
| [$(NpSn)_4S_6$] in **4** | Sn–S<br>Sn–C<br>Sn…Sn<br>S…S | 2.3907(9) – 2.4290(11) Å<br>2.125(4) –2.1454(4) Å<br>3.7795(5) –3.8341(6) Å<br>3.8662(16) – 4.0452(2) Å |
| | S–Sn–S<br>Sn–S–Sn | 106.073(34) – 114.723(36) °<br>103.959(41) –106.149(41) ° |
| [$(NpSn)_4S_6$] in **5** | Sn–S<br>Sn–C<br>Sn…Sn<br>S…S | 2.393(2) – 2.412(2) Å<br>2.122(9) –2.164(9) Å<br>3.7892(7) –3.8128(8) Å<br>3.8781(37) – 4.0333(33) Å |
| | S–Sn–S<br>Sn–S–Sn | 108.218(80) – 115.728(77) °<br>104.479(92) –105.305(83) ° |
| [$(NpSn)_4S_6$] in **6** | Sn–S<br>Sn–C<br>Sn…Sn<br>S…S | 2.3852(2) – 2.418(2) Å<br>2.103(14) –2.120(17) Å<br>3.7631(13) –3.8105(18) Å<br>3.9831(43) – 4.0036(43) Å |
| | S–Sn–S<br>Sn–S–Sn | 107.72(8) – 113.60(8) °<br>103.45(8) –106.45(9) ° |
| [$(NpSi)_4S_6$] in **D** (ref. [2]) | Si–S<br>Si–C<br>Si…Si<br>S…S | 2.111(3) – 2.15(3) Å<br>1.873 (8) –1.876(8) Å<br>3.3474(34) –3.3797(30) Å<br>3.5067(30) – 3.5578(29) Å |
| | S–Si–S<br>Si–S–Si | 111.90(12) – 112.72(13) °<br>104.12(12) –105.64(13) ° |

## 2. Supplementary information on the optical absorption spectroscopy

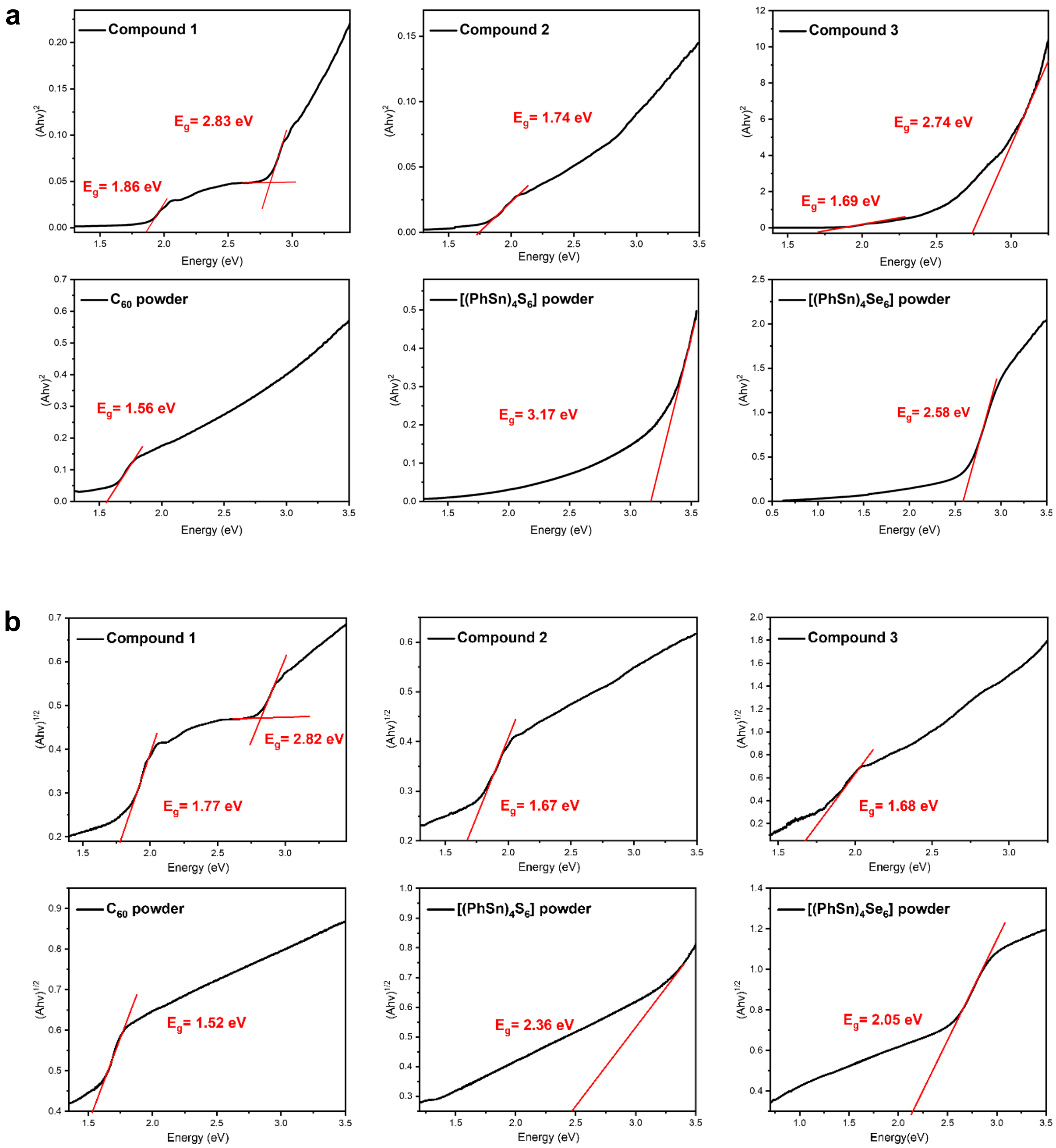


**Supplementary Figure 21 | Tauc plots of compounds 1 – 3, $C_{60}$, $[(PhSn)_4S_6]$ (A), and $[(PhSn)_4Se_6]$ (B). a,** Tauc plots according to $(F(R_\infty)h\nu)^{1/\gamma}$, with $\gamma = ½$, indicative for a direct allowed optical gap. **b,** Tauc plots according to $(F(R_\infty)h\nu)^{1/\gamma}$, with $\gamma = 2$, indicative for an indirect allowed optical gap direct band gap.[refs. 3-5]

### 3. Supplementary information on the nonlinear optical response

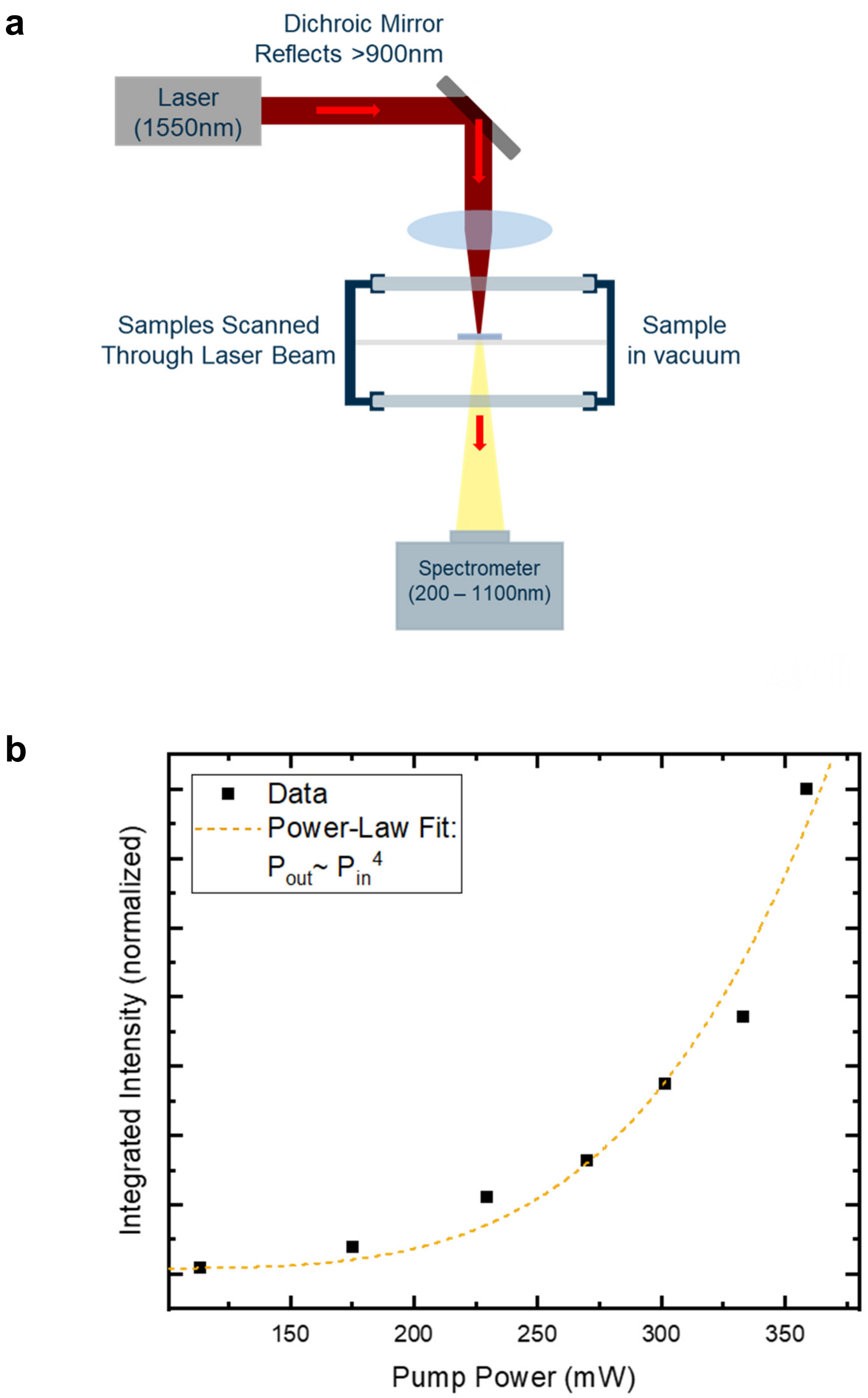


**Supplementary Figure 22 | Setup for measuring the nonlinear optical response and input-output characteristics of the white-light emission. a,** Scheme of the experimental setup for measuring the nonlinear optical response. **b,** The emission spectra for various pump powers are integrated over the whole spectral range of detection. The resulting integrated intensities (black squares) are normalized to the maximum value and plotted over the respective pump power. To underline the nonlinear behavior, a power-law fit (yellow dashed curve) proportional to the fourth power of the input is shown.

## 4. Supplementary information on the first-principles calculations of the electronic properties of the cocrystals

**Supplementary Table 4 | Lattice parameters of the crystalline compounds 1 (in rhombohedral setting), 2 and 3 as calculated within DFT.**

| | Crystal lattice type | | a [Å] | b [Å] | c [Å] | $\alpha$ [°] | $\beta$ [°] | $\gamma$ [°] |
|---|---|---|---|---|---|---|---|---|
| **1** | Rhombohedral | | 19.612 | 19.612 | 19.612 | 87.83 | 87.83 | 87.83 |
| **2** | Monoclinic | | 23.032 | 19.248 | 24.378 | 90 | 90 | 102.01 |
| **3** | Orthorhombic | | 18.643 | 25.131 | 28.992 | 90 | 90 | 90 |

Core···core distances (center-to-center) as well as shortest distances between chalcogenide atoms (S or Se) and the hexagonal faces of neighboring $C_{60}$ molecules are within 1% of the measured values.

In order to estimate the extension of the cluster core, a convex hull's volume of the core is considered. The corresponding results are shown in **Supplementary Table 5**, which demonstrates that the core volume is substantially preserved upon crystallization. The volume of [$(PhSi)_4S_6$], for comparison, is substantially smaller. **Supplementary Table 5** also shows the Si–S, Sn–S as well as Sn–Se bond lengths of the investigated compounds. The average bond length roughly mirrors the sum of the covalent radii of the involved atoms. The difference between maximal and minimal bond length is a measure for the deviation from the ideal tetragonal symmetry of the adamantane-based core, and can thus be interpreted as a core distortion. We observe that the deviation from the tetrahedral symmetry is similar in [$(PhSn)_4S_6$], [$(PhSn)_4Se_6$], and [$(PhSi)_4S_6$]. Upon crystallization, the core distortion is enhanced, in particular for crystal **3** containing Se.

**Supplementary Table 5 | Volume of the {$T_4E_6$} cluster core and T–E bond lengths as calculated by DFT for isolated clusters [$(PhSn)_4S_6$], [$(PhSn)_4Se_6$], and [$(PhSi)_4S_6$] and upon their inclusion in the crystals of compounds 1, 2, and 3.** Minimal, maximal and average T–E bond lengths are given, as well as the difference between smallest and largest length. The latter is a measure for the core distortion.

| | Volume of {$Te_4E_6$}[Å$^3$] | T–E min. [Å] | T–E max. [Å] | T–E avg. [Å] | Diff. [Å] |
|---|---|---|---|---|---|
| [$(PhSn)_4S_6$] | 37.46 | 2.424 | 2.436 | 2.43 | 0.012 |
| [$(PhSn)_4Se_6$] | 44.03 | 2.554 | 2.565 | 2.56 | 0.011 |
| **1** | 37.26 | 2.421 | 2.440 | 2.43 | 0.019 |
| **2** | 37.29 | 2.420 | 2.444 | 2.43 | 0.024 |
| **3** | 43.94 | 2.534 | 2.578 | 2.56 | 0.044 |
| [$(PhSi)_4S_6$] | 25.83 | 2.140 | 2.153 | 2.15 | 0.013 |

The structures of isolated clusters [$(PhSn)_4S_6$] and [$(PhSn)_4Se_6$] are rather similar, with larger bond lengths in [$(PhSn)_4Se_6$] corresponding to the larger covalent radius of Se with respect to S (120 pm vs 105 pm). The analysis of the interatomic distances and angles within [$(PhSn)_4S_6$] and [$(PhSn)_4Se_6$] – as free-standing molecules, as pairs, and as extracted from the crystalline model and the crystal structures of compounds **1**, **2**, and **3** – allows to extrapolate some insight concerning the molecular structures of [$(PhSn)_4S_6$] and [$(PhSn)_4Se_6$]. The interatomic distances are compared in **Supplementary Table 6**.

First of all, we observe that the bond lengths calculated in this work for [$(PhSn)_4S_6$] and [$(PhSn)_4Se_6$] are in very good agreement with previous theoretical studies from ref. [6]. The interatomic distances of the clusters in the calculated crystal structures of **1**, **2**, and **3** are also very close to the experimentally measured values. Second, it can be observed that the interatomic distances calculated for isolated molecules are slightly larger than the corresponding values calculated and measured for the molecules in the crystals, suggesting that the [$(PhSn)_4S_6$] and [$(PhSn)_4Se_6$] clusters experience a small compression in the ordered structure.

**Supplementary Table 6 | Comparison of interatomic distances and angles in cluster core of [(PhSn)$_4$S$_6$] and [(PhSn)$_4$Se$_6$], as calculated by DFT, with experimental values**. The figures are given for free-standing clusters (“Isolated, this work”), for clusters excised from the calculated crystal structures of **1**, **2**, and **3** (“Cocrystal, this work”), for free-standing clusters as computed with slightly different methods in ref. [6] (“Isolated, ref. [6]”), from cluster pairs computed in ref. [6] (“Pair, ref. [6]”). Values determined on compounds **1**, **2**, and **3** (“Exp., this work”) are given for comparison. The dimers considered for the analysis are ones with alternating (stacking) positions of the substituents, very similar to the conformations occurring in the crystal structures of **1** – **3**. All lengths are given in Å, angles are given in °.

| | Isolated, this work) | Cocrystal, this work | Isolated, ref. [6] | Pair, ref. [6] | Exp., this work |
|---|---|---|---|---|---|
| Compound **1** | | | | | |
| Sn–S | 2.424 – 2.435 | 2.422 – 2.446 | 2.440 – 2.450 | 2.438 – 2.451 | 2.395 – 2.411 |
| Sn–C | 2.145 – 2.146 | 2.138 – 2.152 | 2.146 – 2.147 | 2.144 – 2.148 | 2.103 – 2.120 |
| Core···Core | | 6.216 | | 5.949 | 6.192 |
| S–Sn–S | 111.33 – 114.26 | 107.72 – 116.39 | 111.08 – 114.53 | 109.62 – 116.86 | 108.95 – 116.00 |
| Sn–S–Sn | 102.87 – 104.34 | 102.84 – 105.35 | 102.02 – 104.33 | 102.60 – 103.44 | 103.49 – 105.31 |
| | | | | | |
| Compound **2** | | | | | |
| Sn–S | 2.424 – 2.435 | 2.412 – 2.438 | 2.440 – 2.450 | 2.438 – 2.451 | 2.380 – 2.411 |
| Sn–C | 2.145 – 2.146 | 2.142 – 2.146 | 2.146 – 2.147 | 2.144 – 2.148 | 2.105 – 2.166 |
| Core···Core | | 6.459 | | 5.949 | 6.405 |
| S–Sn–S | 111.33 – 114.26 | 107.67 – 116.81 | 111.08 – 114.53 | 109.62 – 116.86 | 107.80 – 116.00 |
| Sn–S–Sn | 102.87 – 104.34 | 103.03 – 106.54 | 102.02 – 104.33 | 102.60 – 103.44 | 103.09 – 104.76 |
| | | | | | |
| Compound **3** | | | | | |
| Sn–Se | 2.554 – 2.565 | 2.535 – 2.576 | n/a | n/a | 2.514 – 2.534 |
| Sn–C | 2.151 – 2.153 | 2.150 – 2.153 | n/a | n/a | 2.110 – 2.126 |
| Core···Core | | 6.459 | n/a | n/a | 6.468 |
| Se–Sn–Se | 112.41 – 115.69 | 109.15 – 118.15 | n/a | n/a | 110.50 – 116.24 |
| Sn–Se–Sn | 100.04 – 101.81 | 100.04 – 102.60 | n/a | n/a | 100.81 – 102.36 |

Moreover, a (rather minor) distortion occurs for the cores of [(PhSn)$_4$S$_6$] and [(PhSn)$_4$Se$_6$] in the crystals, as suggested by the slightly larger deviation between maximal and minimal values. This small distortion with respect to the free-standing molecular clusters is due to the crystalline environment, in particular the presence of C$_{60}$, toluene or THF molecules and adjacent [(PhSn)$_4$S$_6$] or [(PhSn)$_4$Se$_6$] clusters. Isolated clusters and cluster pairs excised from the calculated structure of **1** are depicted in **Supplementary Figure 21.**

The observed changes occur due to the interaction between the substituents and between the cores of neighboring clusters, respectively. As indicated in **Supplementary Table 6**, the core···core distances within compounds **1** and **2** are very close to those of free-standing (calculated) cluster pairs (see ref. [3]), suggesting a relatively strong core···core interactions. As a consequence, the orientations of the phenyl groups tht are closest to each are adjusted.

For compound **2** the situation is similar. However, we observe slightly larger core···core distances and a less pronounced rotation of the substituents. As known from the previous investigations, tetraphenyl hetero-adamantanes are characterized by very low rotational barriers for the substituents, which are thus rather free to rotate and adapt to the environment.

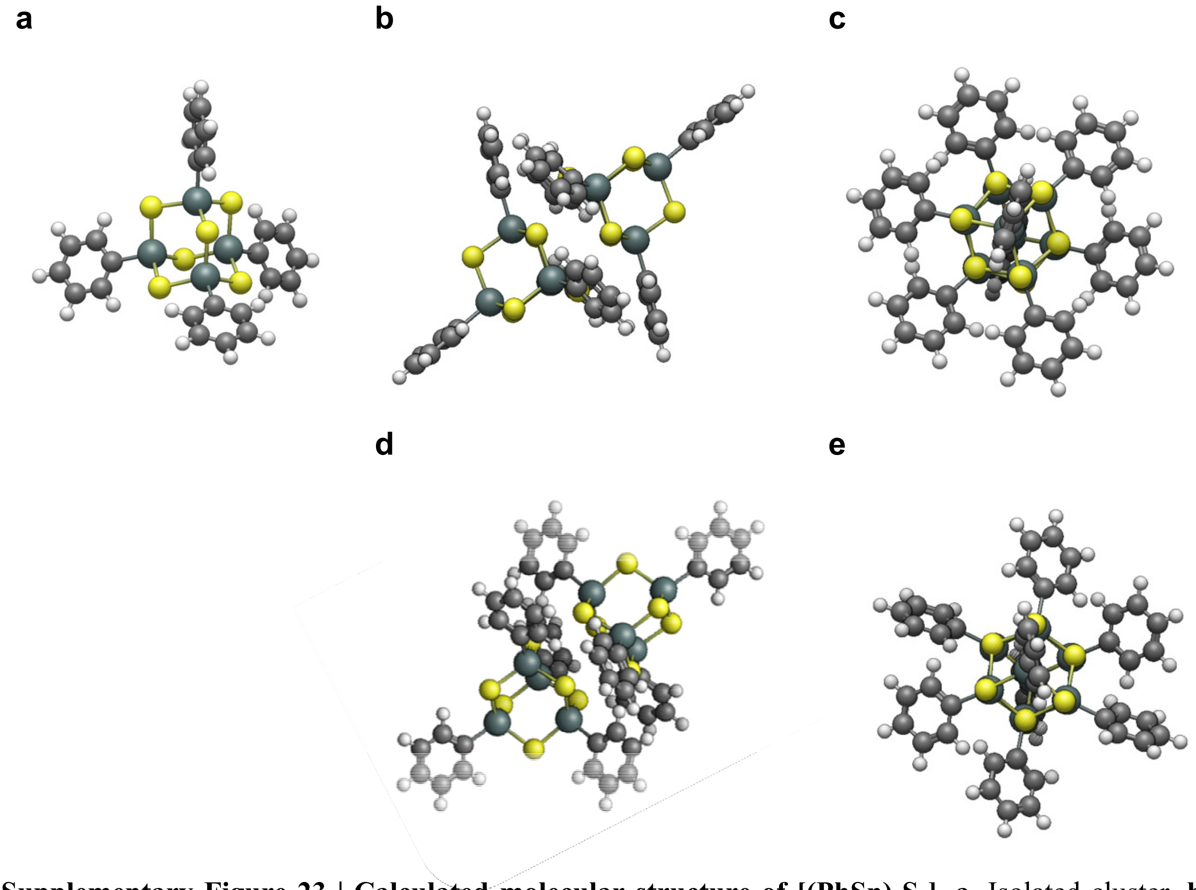


**Supplementary Figure 23 | Calculated molecular structure of [$(PhSn)_4S_6$]. a,** Isolated cluster. **b,** Cluster pair extracted from the calculated crystal structure of **1** in side view. **b,** Cluster pair extracted from the calculated crystal structure of **1** in top view. **d,** Cluster pair extracted from the calculated crystal structure of **2** in side view. **e,** Cluster pair extracted from the calculated crystal structure of **2** in top view. The color coding is the same as in the main document, with S in yellow, Sn in dark green, C in black and H in white.

We wondered whether the analysis of the electrostatic potential allows to draw information about the crystallization tendency and the distribution of the molecular clusters within the crystalline compounds. Therefore, the electrostatic potential was calculated for clusters [$(PhSn)_4S_6$] and [$(PhSn)_4Se_6$] as isolated molecules and within compounds **1** and **3**. **Supplementary Figure 24** illustrates the results on the example of clusters [$(PhSn)_4S_6$] and [$(PhSn)_4Se_6$], as calculated for isolated molecules and as calculated for a cluster within compound **1**.

It can be observed that both the S atoms in the cluster core and the organic substituents (to a lesser extent) correspond to high potential regions, which experience attractive interactions with the low potential regions, also localized in the cluster core. Indeed, in the gas phase they form stacked pairs, in which the core-core interactions clearly dominate the contributions to the cohesive energy.

While the strong and rather isotropic core···core interactions of the isolated molecular clusters leads to a strong tendency for arbitrary aggregation without a suitable cocrystallization template, and thus, to disordered, amorphous compounds, the presence of the $C_{60}$ allows for the formation of ordered, crystalline structures by π-π interactions between their surface and the aromatic organic substituents of the clusters that provide higher-potential regions.

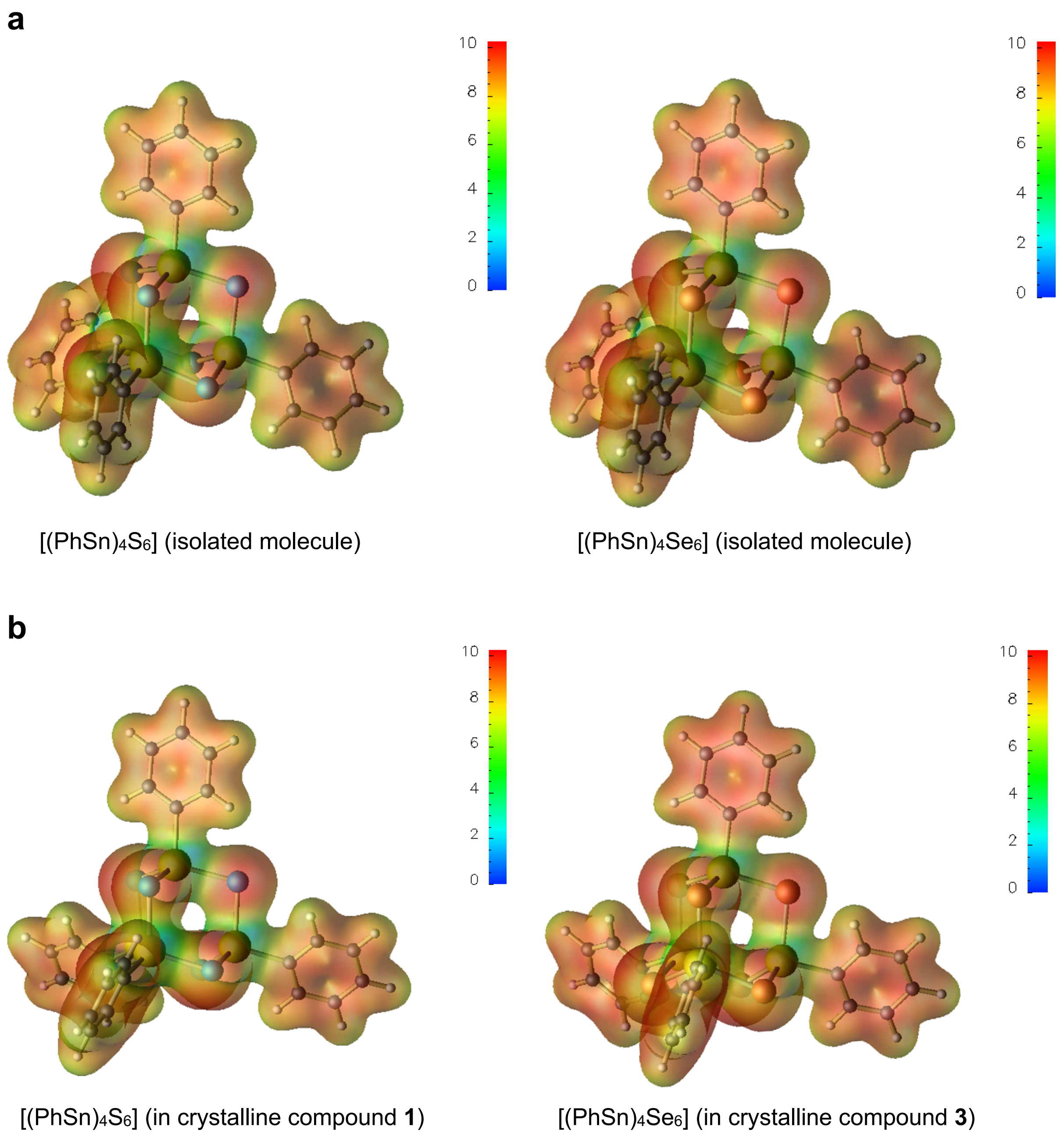


**Supplementary Figure 24 | Molecular electrostatic potentials of [(PhSn)$_4$S$_6$] and [(PhSn)$_4$Se$_6$]. a,** Electrostatic potentials as calculated for an isolated cluster of **[(PhSn)$_4$S$_6$]** (left) and **[(PhSn)$_4$S$_6$]** (right). **b,** Electrostatic potentials as calculated for a cluster within crystalline compound **1** (left) and within crystalline compound **3** (right). The values in the scalebars are relative numbers (in eV). The figures indicate very slight differences in the electrostatic potential of isolated versus cocrystallized clusters only.

**a**

**b**

**c**

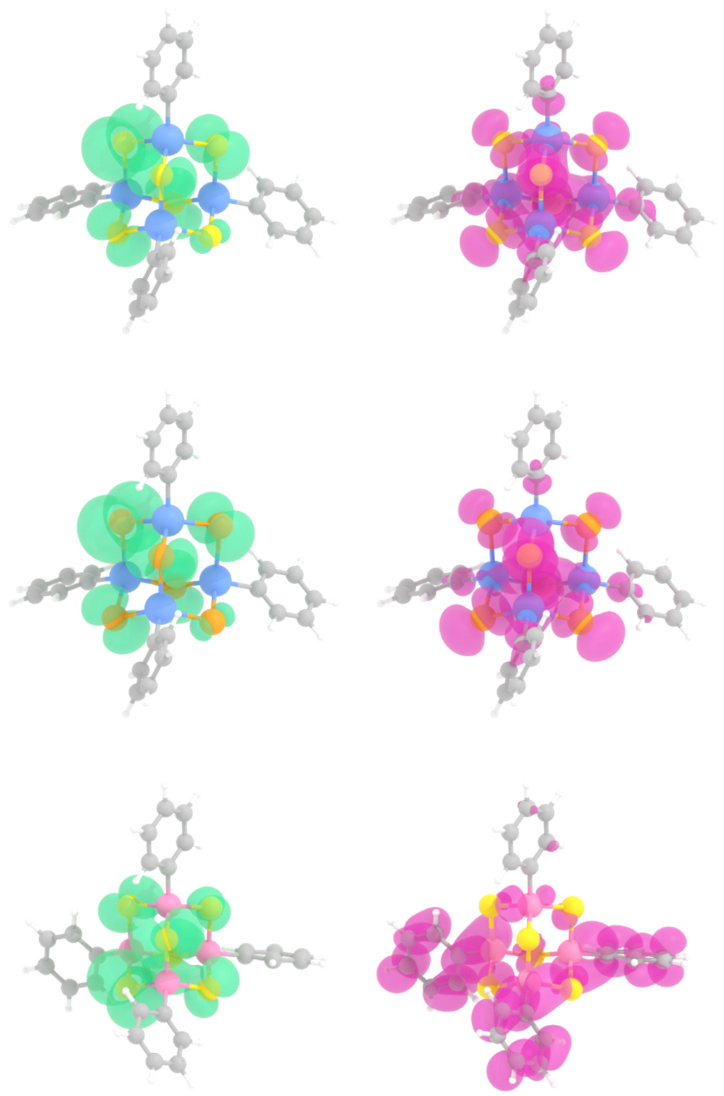

**Supplementary Figure 25 | Illustration of HOMO and LUMO of clusters [(PhT)$_4$E$_6$] (T = Sn, Si; E = S, Se) calculated within DFT-PBE. a,** HOMO (left) and LUMO (right) of [(PhSn)$_4$S$_6$]. **b,** HOMO (left) and LUMO (right) of [(PhSn)$_4$Se$_6$]. **c,** HOMO (left) and LUMO (right) of [(PhSi)$_4$S$_6$]. The HOMO is strongly localized at the S atoms, while the LUMO is less localized at the S atoms and extends to the substituents, especially for T = Si. The isosurface at 0.001 $e^{-3}$ is shown.

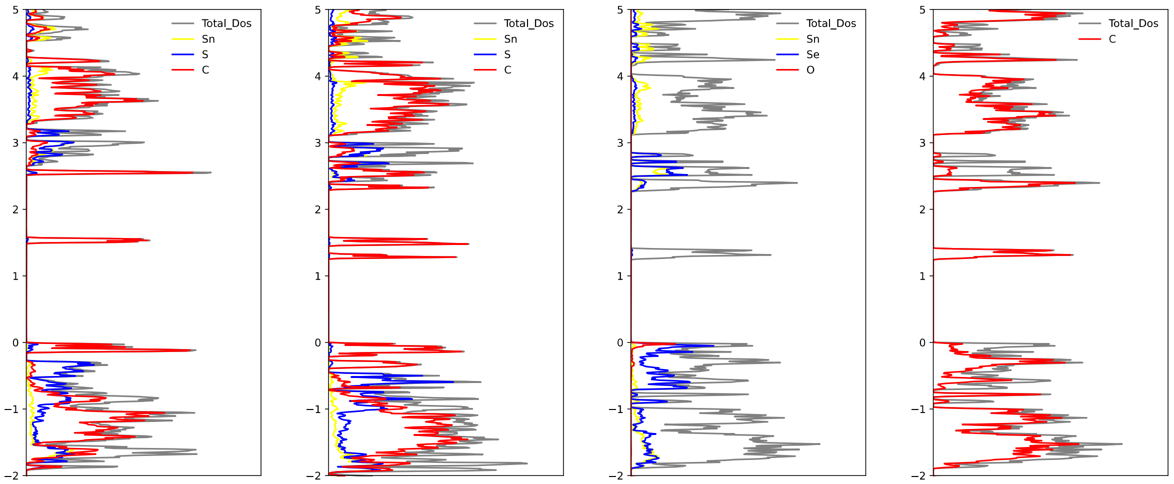


**Supplementary Figure 26 | Density of states and partial density of states calculated within DFT-PBE for compounds 2 and 3.** For the sake of clarity, the DOS of **3** is split into the last two panels. The corresponding graphic for compound **1** is given in **Figure 6f** in the main document.

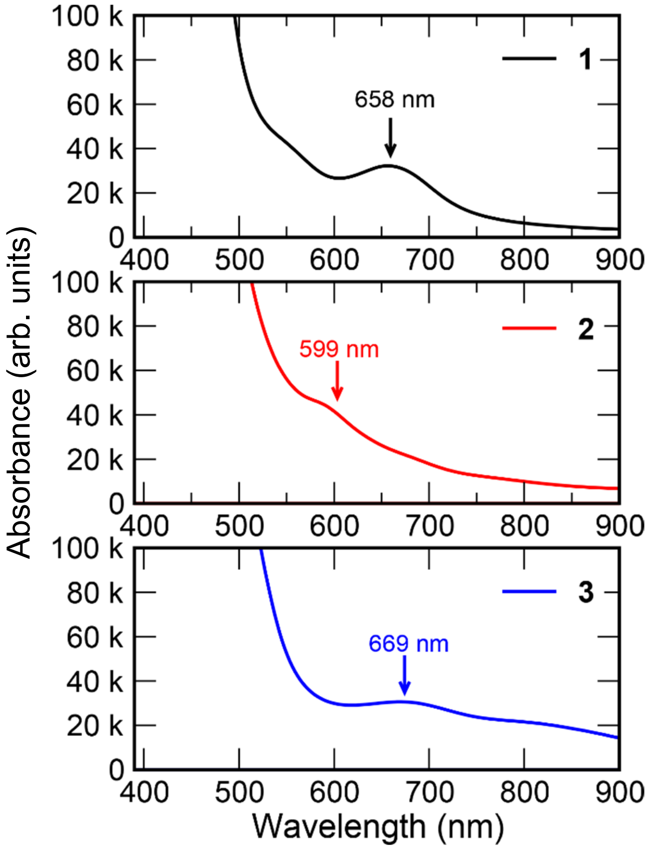


**Supplementary Figure 27 | Optical absorption of 1, 2, and 3.** Absorption coefficient of **1**, **2**, and **3** calculated in the independent particle approximation. The arrows mark spectral signatures related to electronic transitions involving the $C_{60}$ states.

## 5. References


1. Rosemann, N. *et.al.* Organotetrel Chalcogenide Clusters: Between Strong Second-Harmonic and White-Light Continuum Generation*.* *J. Am. Chem. Soc.* **138**, 16224–16227 (2016).

2. Hanau, K. *et al.* Towards Understanding the Reactivity and Optical Properties of Organosilicon Sulfide Clusters. *Angew. Chem. Int. Ed.* **60**, 1176–1186 (2021).

3. Boldish, S. I. *et al.* Optical Band Gaps of Selected Ternary Sulfide Minerals. *Am. Mineral.* **83**, 865–871(1998).

4. Escobedo-Morales, A. *et al.* Automated Method for the Determination of the Band Gap Energy of Pure and Mixed Powder Samples Using Diffuse Reflectance Spectroscopy. *Heliyon*, **5**, e01505–e01505 (2019).

5. Michalow, K. A. *et al.* Synthesis, Characterization and Electronic Structure of Nitrogen-Doped $TiO_2$ Nanopowder. *Catal. Today*, **144**, 7–12 (2009).

6. Schwan, S. *et al.* Insights into molecular cluster materials with adamantane-like core structures by considering dimer interactions. *J. Comput. Chem.* **44**, 843–856 (2023).
